# Perioperative intravenous fluid management in paediatric surgery: a scoping review protocol

**DOI:** 10.1136/bmjopen-2025-112113

**Published:** 2026-02-06

**Authors:** Viviana Leidy Sanchez, Valentina Pinzon Rodas, Ginna Cabra-Bautista, Ivan D Florez, Markus Klimek, Jose A Calvache

**Affiliations:** 1 Department of Anesthesiology, Universidad del Cauca, Popayan, Colombia; 2 Department of Pediatrics, Universidad del Cauca, Popayan, Colombia; 3 Department of Pediatrics, Universidad de Antioquia, Medellín, Colombia; 4 Pediatric Intensive Care Unit, Clínica Las Américas, Medellín, Colombia; 5 School of Rehabilitation Science, McMaster University, Hamilton, Ontario, Canada; 6 Department of Anesthesiology, Erasmus MC, Rotterdam, The Netherlands

**Keywords:** Fluid Therapy, PAEDIATRICS, Paediatric anaesthesia, PAEDIATRIC SURGERY

## Abstract

**Abstract:**

**Introduction:**

Intravenous fluids are essential components of perioperative care, supporting intravascular volume, acid–base balance and electrolyte homeostasis. Despite extensive research in adult surgical populations, paediatric-specific evidence remains limited, and clinical practice frequently relies on extrapolated adult-based recommendations. This gap is particularly relevant in paediatric non-cardiac surgery, where fluid choice may influence key physiological outcomes such as acid–base status, electrolyte balance, renal function and haemodynamic stability. Given the heterogeneity of study designs, perioperative phases, age groups and reported outcomes in the paediatric literature, a comprehensive synthesis of the existing evidence is needed before a systematic review can be undertaken.

**Methods and analysis:**

We will conduct this scoping review following the methodological guidance of the Joanna Briggs Institute Manual for Evidence Synthesis, and the reporting will adhere to the Preferred Reporting Items for Systematic Reviews and Meta-Analyses extension for Scoping Reviews guideline.

This scoping review will map existing evidence on perioperative intravenous fluid management in paediatric patients (<18 years) undergoing elective non-cardiac surgery in outpatient and inpatient settings. Eligible study designs will include randomised trials, observational studies and systematic reviews. A comprehensive search will be developed with a medical librarian and applied to MEDLINE (PubMed), Ovid, Embase, Web of Science, CENTRAL, Google Scholar and ClinicalTrials.gov, with no date restrictions and limited to English, Spanish and German.

Eligibility is framed using participants, concept and context: paediatric patients (<18 years) undergoing non-cardiac surgery; concepts related to preoperative fasting/replacement period, intraoperative period and postoperative period up to 24 hours, intravenous fluid management for maintenance/replacement; and hospital/outpatient surgical settings. Study selection and data charting will follow established scoping review methodology. Data will be synthesised descriptively using narrative and tabular formats. No meta-analysis or formal risk-of-bias appraisal is planned, consistent with scoping review methodology.

**Ethics and dissemination:**

This scoping review involves no primary data collection and relies exclusively on published literature; therefore, formal ethical approval is not required. The protocol received administrative approval from the Comité de Ética para la Investigación Científica of Universidad del Cauca (approval no. 6553, 11 June 2025). Findings will be disseminated through peer-reviewed publications, conference presentations and targeted communication with paediatric anaesthesia and surgical communities.

**STRENGTHS AND LIMITATIONS OF THIS STUDY**

- This protocol follows the Preferred Reporting Items for Systematic Reviews and Meta-Analyses extension for Scoping Reviews guideline and the Joanna Briggs Institute Manual, ensuring methodological transparency and reproducibility.
- A comprehensive search strategy will be implemented across multiple major databases (MEDLINE, Embase, Cochrane CENTRAL, Web of Science, ClinicalTrials.gov, Google Scholar), with support from a medical librarian.
- Independent and duplicate screening and data extraction will be conducted to reduce bias.
- This review aims to map the breadth of available evidence on perioperative fluid management in paediatric non-cardiac surgery, a field characterised by heterogeneous study designs and limited randomised data.
- This review includes the restriction to studies published in English, Spanish and German and the absence of a formal risk-of-bias assessment, consistent with scoping review methodology.

## Introduction

 Perioperative fluid management aims to maintain normal fluid balance and electrolyte levels, typically achieved through the administration of intravenous fluids in hospitals and during surgery.^1^ Surgery triggers physiological and hormonal responses that require careful management, with fluid and electrolyte balance being a central component.^2 3^

During the perioperative period, intravascular volume can be affected by multiple factors, including fasting duration, type and length of surgery, sensible and insensible losses during the surgical procedure, the volume of fluids administered for drug and/or anaesthetic dilution and individual comorbidities that may predispose to fluid redistribution into the extravascular compartment.^4^ These factors must be carefully considered when administering intravenous fluids to replace losses and maintain basal fluid requirements.^5^

In paediatric patients, these challenges are more pronounced due to physiological differences in plasma volume regulation, immaturity of neurohormonal systems (predisposing them, eg, to more frequent episodes of hypoglycaemia), and acid–base balance.^2 3 5^ Fluid and electrolyte management in children therefore requires a comprehensive understanding of their physiology and organ immaturity, which varies depending on the stage of growth.^1 3^ Neonates, in particular, exhibit physiologic and metabolic responses that differ substantially from those of infants, children and adolescents, including higher susceptibility to hypoglycaemia, hyperglycaemia during surgical stress and overall greater perioperative morbidity. For these reasons, neonatal data must be considered separately. These age-related differences make it inappropriate to apply general adult recommendations for intravenous fluid use in paediatric patients undergoing surgery without considering their particularities.

Intravenous fluids have been initially classified as crystalloids or colloids.^5^ Buffered crystalloids, with plasma-like electrolytes, help maintain acid–base balance, while non-buffered solutions (eg, 0.9% saline) are linked to hyperchloraemic acidosis, kidney injury,^6^ endothelial damage, oedema and adverse haemodynamics.^1 2 7 8^ Colloids, by increasing oncotic pressure, are mainly used for rapid intravascular volume expansion.^2 5 9 10^ Studies comparing different types of fluids in terms of mortality and secondary outcomes have been more extensively conducted in adult populations, particularly in critically ill patients and in perioperative care.^11^ Consequently, several systematic reviews and meta-analyses have provided strong evidence-based recommendations favouring the use of balanced solutions in adults.^12^ However, there is a lack of similar high-quality evidence for the paediatric population where research remains limited and where adult guidelines have been extrapolated to this group.^2 5^

Given the limited and heterogeneous nature of the available paediatric evidence, a systematic review is currently not yet feasible. The literature spans multiple study designs, varying perioperative periods, mixed age groups and inconsistent outcome measures, with many practices still extrapolated from adult research. A scoping review is therefore the most appropriate approach to map how perioperative intravenous fluids have been studied in children, to describe the range and characteristics of existing evidence, and to identify gaps that must be addressed before a systematic review can be undertaken.

### Research questions

The objective of this scoping review is to gather and describe the current evidence on the use of preoperative fasting/replacement period, intraoperative period and postoperative period up to 24 hours in paediatric patients undergoing non-cardiac surgery. The review focuses on identifying the types of fluids most commonly used, their physiological impact, and the benefits and risks associated with each, including outcomes such as electrolyte disturbances, acid–base imbalances, haemodynamic effects, renal function, glycaemic status and postoperative complications across different paediatric age groups. Neonates will be analysed separately due to their unique physiological characteristics.

The scoping review will address the following questions:

1. What are the types of intravenous fluids used in perioperative management of paediatric patients undergoing non-cardiac surgery?
2. What benefits and adverse effects are reported for different intravenous fluid types, including balanced versus non-balanced crystalloids, synthetic versus natural colloids and combined dextrose–crystalloid solutions, in relation to clinical outcomes such as electrolyte balance, acid–base homeostasis, renal function, glycaemic status and haemodynamic stability?
3. How is intravenous fluid therapy managed across different perioperative phases (preoperative fasting/replacement period, intraoperative period and postoperative period up to 24 hours), and where do evidence gaps remain in paediatric perioperative fluid management?

## Methods and analysis

This scoping review protocol is conducted following the methodological guidance of the Joanna Briggs Institute (JBI) Manual for Evidence Synthesis^13^ and is reported in accordance with the Preferred Reporting Items for Systematic Reviews and Meta-Analyses extension for Scoping Reviews (PRISMA-ScR) guidelines.^14^

We selected a scoping review design rather than a systematic review because the available paediatric evidence is methodologically diverse, sparse and insufficiently comparable to support quantitative synthesis or formal risk-of-bias appraisal. Consistent with JBI guidance, the primary aim of this scoping review is to ‘map the existing evidence’, providing a comprehensive overview of what is currently known about perioperative fluid strategies in children. By charting how these interventions have been studied—rather than evaluating their effectiveness—we seek to clarify the scope, characteristics and gaps within the literature and to determine whether the evidence base is robust enough to warrant a future systematic review.

### Definition of perioperative period

For the purposes of this review, the term *perioperative* refers to the continuum that includes:

1. The preoperative fasting and replacement period.
2. The intraoperative period.
3. The early postoperative period, defined as the first 24 hours after completion of surgery.

This definition will be applied consistently throughout the review.

### Eligibility criteria

The review will explore current literature on the use of intravenous fluids in the perioperative care of paediatric patients including both balanced and unbalanced crystalloid solutions, natural and synthetic colloids, and, where relevant, dextrose-containing solutions across different age groups.

We will consider randomised controlled trials (RCTs), systematic reviews and observational studies such as cohort, case–control and cross-sectional designs comparing different types of intravenous fluids, excluding studies in animals and case reports. We will use the participants, concept and context criteria to define eligibility criteria.^13^

1. Participants: paediatric patients, from neonates to individuals under 18 years of age, undergoing non-cardiac surgery.
2. Concept: intravenous fluid management in the perioperative period, specifically for replacement and maintenance purposes. Included studies must evaluate outcomes related to intravenous fluid administration. We will exclude hyperosmolar fluids used for specific therapeutic purposes such as intracranial hypertension and cardioplegia solutions used in cardiac surgeries, as they are not intended mainly for fluid replacement.
3. Additionally, dextrose-containing solutions administered during induction, particularly in neonates and infants, will be included when they are used as part of the perioperative intravenous fluid strategy (for maintenance or replacement) or when their physiological effects (eg, glycaemic stability, acid–base status, electrolyte balance) are evaluated in the context of perioperative fluid management. Solutions used exclusively for drug dilution will not be considered unless they constitute part of the fluid management intervention.
4. Context: we will include both outpatient and hospitalised patients undergoing non-cardiac surgery, but exclude those in critical care settings, as well as patients with acute severe conditions requiring massive fluid replacement (eg, trauma with emergency surgery) or those who are haemodynamically unstable.

These exclusions were applied because resuscitation pathways, cardiac bypass perfusion and neurotherapeutic osmotherapy represent fundamentally different fluid strategies from perioperative maintenance and replacement and therefore fall outside the scope of this review.

The primary outcomes of interest will include perioperative fluid balance, haemodynamic stability, electrolyte disturbances, acid–base status, coagulopathy or alterations in coagulation parameters (when synthetic colloids are evaluated), incidence of postoperative complications and overall clinical outcomes. Secondary outcomes will assess comparative efficacy between fluid types, adverse effects such as hyperchloraemic acidosis, acute kidney injury and oedema formation, and the influence of fluid choice on recovery parameters, including length of hospital stay and postoperative morbidity.

### Search strategy

A detailed search strategy will be prepared with assistance from a professional librarian and is available in online supplemental file 1. Erasmus MC University Medical Library will develop and update the search strategies. A structured search will be conducted in the following sources: Medline (Pubmed), Ovid, Embase, Web of Science Core Collection, Cochrane Central Register of Controlled Trials and Google Scholar. All searches will be performed through October 2025 and will be limited to studies involving humans. The full electronic search strategy is provided in online supplemental materials. Publications such as conference abstracts, editorials and notes will be excluded. ClinicalTrials.gov will also be searched. Furthermore, the reference lists for all relevant literature will be manually examined. Relevant trials will be included regardless of publication year. Only studies published in Spanish, English and German will be considered.

Although the supplementary search strings include RCT-sensitive lines for transparency, the final search strategy used for the review will not apply design filters. No randomised trial filters or methodological limits will be used in the operational search, ensuring full capture of observational, descriptive and non-experimental paediatric surgical fluid studies. A broad, unrestricted line will be included to avoid excluding relevant paediatric cohorts, which represent the majority of available evidence in this field.

### Screening and selection of studies

Three independent reviewers (VLS, VPR and GC-B) will screen titles and abstracts in duplicate and assess the full texts of all relevant studies for inclusion by using Covidence.^15^ In the first stage, two independent reviewers will screen the titles and abstracts of all potentially eligible publications. In the second stage, full-text articles meeting the initial criteria will be assessed independently by the same reviewers against the predefined eligibility criteria. Any discrepancies between reviewers will be resolved by consensus or by involving a third review author (JAC). The study selection process will be presented using a PRISMA-ScR flow diagram.

### Data extraction and management

Data extraction will be conducted using a predefined data extraction template. A pilot version of the extraction form will be tested to ensure accuracy and feasibility, with modifications made as needed based on the retrieved data. Full-text articles will be independently extracted by two researchers to minimise bias and enhance reliability. Any discrepancies will be resolved through discussion, or if necessary, by consulting a third reviewer. Study exclusions will be systematically documented. A PRISMA-ScR flow diagram will be used to illustrate the study selection process.^16^ If a required data item is not available in a publication, it will be recorded as ‘not reported’ and left blank in the data charting tables. Potential conflicts of interest will be considered, and verification of the availability of a published protocol for each study will be performed.

Data will be charted using a predefined and piloted data extraction form. The preliminary data extraction instrument is provided in online supplemental table S1.

### Data analysis

Data will be synthesised descriptively to provide a comprehensive overview of the intravenous fluid management strategies used in the perioperative care of paediatric patients undergoing non-cardiac surgery. Results will be categorised according to the type of intravenous fluids administered (balanced vs non-balanced crystalloids, natural and synthetic colloids and dextrose-containing solutions), their indications, physiological effects and the clinical outcomes reported.

In accordance with scoping review methodology, we will not compare outcomes across studies. Instead, we will systematically map the outcomes reported in each study—such as fluid balance, haemodynamic parameters, electrolyte or acid–base disturbances, acute kidney injury, oedema, coagulopathy or coagulation abnormalities (particularly in studies evaluating synthetic colloids) and length of stay—and describe the methods used to measure these outcomes.

To organise heterogeneous data and facilitate interpretation, findings will be presented through narrative synthesis and summary tables. When available, results will be stratified by predefined descriptors, including age groups (neonate, infant, schoolchild, child, adolescent), surgical context (ambulatory vs hospitalised settings), type of surgery and fluid type (balanced crystalloids, 0.9% saline, colloids and dextrose-containing solutions).

Neonates will be analysed and reported as a distinct group, given their unique physiological characteristics, markedly different perioperative risk profile and specific fluid and glucose regulation patterns that are not generalisable to older paediatric cohorts.

Consistent with scoping review methodology, we will not perform a formal risk-of-bias appraisal. We acknowledge that the absence of quality assessment limits the ability to draw conclusions on the effectiveness or comparative safety of the interventions described.

Finally, the review will identify gaps in the existing evidence to clarify where further research is required and to inform the design of future studies. Rather than generating clinical recommendations, this scoping review will provide an evidence map describing the scope, depth and characteristics of current research, thereby supporting priorities for subsequent systematic reviews and primary investigations in paediatric perioperative fluid management.

### Patient and public involvement

Patients and members of the public were not involved in the formulation of the research question, the development of the review methods, the selection or appraisal of evidence, or the interpretation and dissemination of findings. This scoping review synthesises information exclusively from publicly available published sources, and no primary data were collected.

## Discussion

This scoping review aims to offer the currently available evidence of how intravenous fluids are used in paediatric non-cardiac surgery, shedding light on both established practices and areas where evidence is still lacking. By compiling and synthesising data from various studies, we hope to provide a more comprehensive understanding of the benefits and risks associated with different fluid types, particularly buffered versus non-buffered crystalloids, and their effects on key physiological parameters such as acid–base balance, electrolyte stability, renal function and haemodynamic response.

One of the principal findings expected from this review is the identification of patterns in current practice across various age groups and clinical contexts. We anticipate clarifying which fluid types are most used, under what circumstances and what clinical outcomes are associated with each. This synthesis may also help delineate potential risks and benefits specific to fluid composition, particularly regarding hyperchloraemia, acidosis and renal function, which have been a concern with certain solutions like 0.9% saline.

A major strength of this review lies in its comprehensive scope, which includes a wide range of study types and focuses on a population that has historically been underrepresented in clinical fluid research. As a scoping review, this study provides a comprehensive mapping of the evidence, although it does not include a formal quality appraisal.

This review will help clarify areas where further investigation is needed in paediatric perioperative fluid management and provide an evidence map to inform future systematic reviews and guide priorities for primary studies. This scoping review is not intended to evaluate effectiveness, support or provide clinical recommendations.

### Ethics and dissemination

As this study is a scoping review of existing literature, it does not involve primary data collection. This protocol received ethics approval from the ethics committee ‘Comité de Ética para la Investigación Científica at Universidad del Cauca’ (Popayán, Colombia), under protocol number 6553, in its second review session dated 11 June 2025. Since the review relies solely on published data and does not involve human participants or the collection of individual-level information, informed consent was not required.

Results will be disseminated through peer-reviewed publications, conference presentations and targeted communication to paediatric anaesthesia and surgery services. Data-charting templates and online supplemental materials will be made available as part of the final publication or on reasonable request. In addition, the protocol will be registered on the Open Science Framework to enhance transparency and reproducibility.

## Supplementary material

10.1136/bmjopen-2025-112113 online supplemental file 1
