# Supplementary material for: Perioperative intravenous fluid management in paediatric surgery: a scoping review protocol
Source: BMJ Open. 2026 Feb 6;16(2):e112113. doi: 10.1136/bmjopen-2025-112113 (PMC12887505; doi:10.1136/bmjopen-2025-112113)
Supplement: online supplemental file 1 [file bmjopen-16-2-s001.docx]

**Supplementary material**

**Search strategy**

**Supplementary methods – Electronic search strategy**

**Embase**

(colloid/mj/de OR hydrocolloid/mj/de OR crystalloid/mj/de OR 'plasma substitute'/mj/exp OR 'infusion fluid'/mj/exp OR 'fluid therapy'/mj/de OR 'fluid resuscitation'/mj/de OR buffer/mj/de OR bicarbonate/mj/de OR (colloid* OR hydrocolloid* OR crystalloid* OR lactated OR ((plasma) NEAR/3 (substit* OR fresh-frozen)) OR ((infusion OR therap* OR treatm* OR intravenous OR replacement OR resuscitation OR balanced OR non-balanced OR management* OR administration* OR intraoperativ* OR perioperativ* OR intra-operativ* OR peri-operativ*) NEAR/3 (fluid*)) OR ringer* OR hartmann* OR buffer* OR bicarbonate* OR electrolyte* OR sodium-chloride OR saline OR NaCl):ti) AND (surgery/exp OR surgery:lnk OR 'intraoperative period'/de OR 'surgical patient'/de OR (surger* OR surgical* OR operation* OR resect* OR arthroplast* OR transplant* OR hysterectom* OR intraoperativ* OR perioperativ* OR intra-operativ* OR peri-operativ*):ab,ti,kw) AND ('Controlled clinical trial'/exp OR 'Crossover procedure'/de OR 'Double-blind procedure'/de OR 'Single-blind procedure'/de OR (random* OR factorial* OR crossover* OR (cross NEXT/1 over*) OR placebo* OR ((doubl* OR singl*) NEXT/1 blind*) OR assign* OR allocat* OR volunteer* OR trial OR groups):ab,ti,kw) NOT ([Conference Abstract]/lim OR [Conference Review]/lim) NOT ([animals]/lim NOT [humans]/lim) NOT (note/de) NOT ('editorial'/de)

**Medline**

(*Colloids/ OR exp *Crystalloid Solutions/ OR *Plasma Substitutes/ OR *Infusions, Intravenous/ OR *Fluid Therapy/ OR exp *Buffers/ OR *Bicarbonates/ OR (colloid* OR hydrocolloid* OR crystalloid* OR lactated OR ((plasma) ADJ3 (substit* OR fresh-frozen)) OR ((infusion OR therap* OR treatm* OR intravenous OR replacement OR resuscitation OR balanced OR non-balanced OR management* OR administration* OR intraoperativ* OR perioperativ* OR intra-operativ* OR peri-operativ*) ADJ3 (fluid*)) OR ringer* OR hartmann* OR buffer* OR bicarbonate* OR electrolyte* OR sodium-chloride OR saline OR NaCl).ti.) AND (exp General Surgery/ OR exp Surgical Procedures, Operative/ OR surgery.fx. OR exp Intraoperative Period/ OR Perioperative Period/ OR (surger* OR surgical* OR operation* OR resect* OR arthroplast* OR transplant* OR hysterectom* OR intraoperativ* OR perioperativ* OR intra-operativ* OR peri-operativ*).ab,ti,kf.) AND (exp Controlled Clinical Trial/ OR Cross-Over Studies/ OR Double-Blind Method/ OR Single-Blind Method/ OR (random* OR factorial* OR crossover* OR (cross ADJ over*) OR placebo* OR ((doubl* OR singl*) ADJ blind*) OR assign* OR allocat* OR volunteer* OR trial OR groups).ab,ti,kf.) NOT (exp Animals/ NOT Humans/) NOT (congres* OR abstract*).pt. NOT (Comment.pt.) NOT (editorial).pt.

**Cochrane**

((colloid* OR hydrocolloid* OR crystalloid* OR lactated OR ((plasma) NEAR/3 (substit* OR fresh-frozen)) OR ((infusion OR therap* OR treatm* OR intravenous OR replacement OR resuscitation OR balanced OR non-balanced OR management* OR administration* OR intraoperativ* OR perioperativ* OR intra-operativ* OR peri-operativ*) NEAR/3 (fluid*)) OR ringer* OR hartmann* OR buffer* OR bicarbonate* OR electrolyte* OR sodium-chloride OR saline OR NaCl):ti) AND ((surger* OR surgical* OR operation* OR resect* OR arthroplast* OR transplant* OR hysterectom* OR intraoperativ* OR perioperativ* OR intra-operativ* OR peri-operativ*):ab,ti,kw) NOT ("conference abstract":kw OR Trial registry record:pt)

**Web of Science**

TI=(colloid* OR hydrocolloid* OR crystalloid* OR lactated OR ((plasma) NEAR/2 (substit* OR fresh-frozen)) OR ((infusion OR therap* OR treatm* OR intravenous OR replacement OR resuscitation OR balanced OR non-balanced OR management* OR administration* OR intraoperativ* OR perioperativ* OR intra-operativ* OR peri-operativ*) NEAR/2 (fluid*)) OR ringer* OR hartmann* OR buffer* OR bicarbonate* OR electrolyte* OR sodium-chloride OR saline OR NaCl) AND TS=((surger* OR surgical* OR operation* OR resect* OR arthroplast* OR transplant* OR hysterectom* OR intraoperativ* OR perioperativ* OR intra-operativ* OR peri-operativ*) AND (random* OR factorial* OR crossover* OR (cross NEAR/1 over*) OR placebo* OR ((doubl* OR singl*) NEAR/1 blind*) OR assign* OR allocat* OR volunteer* OR trial OR groups) NOT ((animal* OR rat OR rats OR mouse OR mice OR murine OR dog OR dogs OR canine OR cat OR cats OR feline OR rabbit OR cow OR cows OR bovine OR rodent* OR sheep OR ovine OR pig OR swine OR porcine OR veterinar* OR chick* OR zebrafish* OR baboon* OR nonhuman* OR primate* OR cattle* OR goose OR geese OR duck OR macaque* OR avian* OR bird* OR fish*) NOT (human* OR patient* OR women OR woman OR men OR man))) NOT DT=(Meeting Abstract OR Meeting Summary) NOT DT=(Note) NOT DT=(Editorial Material)

**Google Scholar**

colloid|crystalloid|lactated|"plasma substition"|"infusion fluid"|"fluid therapy|replacement"|ringer|hartmann|buffer surgery|surgical|operation "randomized|randomised controlled trial"|RCT -animal -mouse -mice -rat -rats

colloid|crystalloid|lactated|'plasma substition'|'infusion fluid'|'fluid therapy|replacement'|ringer|hartmann|buffer surgery|surgical|operation 'randomized|randomised controlled trial'|RCT -animal -mouse -mice -rat -rats

**Table S1. Preliminary data extraction instrument**

| **Scoping Review Details** | |
| --- | --- |
| **Scoping Review title:** | Perioperative Intravenous Fluid Management in Pediatric Surgery: A Scoping Review |
| **Review objective/s:** | To explore and describe the types of intravenous fluids used in the perioperative management of pediatric patients undergoing non-cardiac surgery, assessing their benefits, adverse effects, and associated outcomes across different pediatric age groups |
| **Review question/s:** | What are the current practices, benefits, risks, and clinical outcomes associated with perioperative intravenous fluid management in pediatric patients undergoing non-cardiac surgery?   - types of intravenous fluids - reported benefits and adverse effects - intravenous fluid therapy across different perioperative phases |
| **Inclusion/Exclusion Criteria** | |
| **Population** | Pediatric patients, from neonates to individuals under 18 years of age, undergoing non-cardiac surgery |
| **Concept** | Intravenous fluid management in the perioperative period, specifically for replacement and maintenance purposes.  **excludes:**  Hyperosmolar fluids used for specific therapeutic purposes such as intracraneal intervention.  Cardioplegia solutions used in cardiac surgeries, as they are not intended for fluid replacement. |
| **Context** | Hospitalized and outpatient pediatric patients undergoing elective non-cardiac surgery.  **excludes:**  Patients in critical or intensive care units.  Patients with acute severe conditions requiring high fluid replacement (e.g., trauma patients undergoing emergency surgery) or hemodynamically unstable. |
| **Types of evidence source** | Randomized controlled trials  Systematic reviews and meta-analyses  Observational studies |
| **Evidence source Details and Characteristics** | |
| **Citation details (e.g. author/s, date, title, journal, volume, issue, pages)** |  |
| **Country** |  |
| **Context** |  |
| **Participants (details e.g. age/sex and number)** |  |
| **Details/Results extracted from source of evidence (in relation to the concept of the scoping review)** | |
