# Supplementary material for: Perioperative intravenous fluid management in paediatric surgery: a scoping review protocol
Source: BMJ Open. 2026 Feb 6;16(2):e112113. doi: 10.1136/bmjopen-2025-112113 (PMC12887505; doi:10.1136/bmjopen-2025-112113)
Supplement: Supplementary file 2 [file bmjopen-2025-112113.reviewer_comments.pdf]

PEER REVIEW HISTORY

BMJ Open publishes all reviews undertaken for accepted manuscripts. Reviewers are asked to complete a checklist review form and are provided with free text boxes to elaborate on their assessment. These free text comments are reproduced below.

ARTICLE DETAILS

Title (Provisional)

PERIOPERATIVE INTRAVENOUS FLUID MANAGEMENT IN PEDIATRIC SURGERY: SCOPING REVIEW PROTOCOL

Authors

Sanchez, Viviana Leidy; Pinzon Rodas, Valentina; Bautista, Ginna; Florez, Ivan D; Klimek, Markus; Calvache, Jose Andres

---

VERSION 1 - REVIEW

**Reviewer**	**1**
**Name**	**Withington, Davinia**
**Affiliation**	**McGill University Faculty of Medicine, Anesthesiology**
**Date**	**01-Nov-2025**
**COI**	**None**

This is a very useful topic to investigate. However, I am finding it difficult to determine why the authors chose a scoping review vs a systematic review. There seems to be an overlap in the aims between former and latter type of investigation.

A scoping review will provide an overview of the literature, which you do, and identify pertinent concepts and gaps in the current research. The protocol appears to be going beyond this by looking in more depth into outcomes. Also the protocol states that the results may improve perioperative outcomes and decrease complications but it would be difficult to achieve this with a Scoping Review which lacks the Quality Appraisal which would be part of a systematic review.

Please clarify which type of investigation is planned and why this was chosen and, if a scoping review, would the investigators move on to a Systematic Review later? The conclusion that a scoping review can contribute to the development of specific clinical guidelines and improve care is overstating the case since a full appraisal of study outcomes is not possible for this study format.

**Reviewer**	**2**

**Name**	**McCarthy, Kevin**
**Affiliation**	**Trinity College**
**Date**	**06-Nov-2025**
**COI**	**None**

Thank you for proposing to undertake this work. Fasting guidelines and peri-operative fluid management in children have undergone significant change over the last decade and some historical and dogmatic practices may no longer apply.

I have two comments:

1. Neonates are physiologically distinct and should be analysed and reported separately. This has been the practice of large-scale observational studies of paediatric practice (e.g. NECTARINE is a separate study from APRICOT) and the NAP-7 National Audit Project in the UK reported a mortality risk that was an order of magnitude higher in neonates than in children. They are also more prone to hypoglycaemia (and hyperglycaemia as part of a surgical stress responses), therefore considerations and recommendations will be specific to this group in a way that is not generalisable to the other paediatric cohorts (infants, children, and adolescents).

2. If synthetic colloids are to be included, then coagulopathy and impact upon coagulation should be included as an outcome of interest.

**Reviewer**	**3**
**Name**	**Reysner, Malgorzata**
**Affiliation**	**Poznan University of Medical Sciences**
**Date**	**06-Nov-2025**
**COI**	**None**

Thank you for submitting this protocol on perioperative intravenous fluid management in pediatric non-cardiac surgery. The topic is well chosen: perioperative fluids in children are still guided largely by extrapolation from adult data, and mapping what actually exists in the literature is valuable and fits well with the aims of a scoping review.

I have a few suggestions to improve clarity and alignment with PRISMA-ScR/JBI, mostly editorial and clarificatory.

Rationale and objectives

The introduction clearly explains why adult data cannot simply be transferred to children (physiological differences, risk of electrolyte/acid–base disturbances, immature regulation). This is good.

In the “Research objectives” and “Research questions” sections there is some overlap and partial repetition. Consider condensing into: (a) overarching objective (to map IV fluids used perioperatively in pediatric non-cardiac surgery) and (b) 3–4 explicit scoping questions (types of fluids; benefits/adverse effects; management across perioperative phases; gaps).

Scope and PCC elements

Your PCC is appropriate for a scoping review: P – pediatric patients <18 y with non-cardiac surgery; C – IV fluid management for maintenance/replacement perioperatively; C – hospital or ambulatory surgical setting.

You exclude critical care, emergency trauma with massive resuscitation, cardioplegia, and hyperosmolar therapeutic fluids — this is sensible, but please say explicitly why these are excluded (i.e. they belong to resuscitation/neurological or cardiac pathways rather than perioperative maintenance).

You may want to clarify what you will do with peri-induction dextrose-containing solutions in neonates/infants (you mention them, so specify that they will be included when used as part of perioperative fluid strategy).

Perioperative time windows

You often write “perioperative (pre/intra/early postoperative)” but in other places you say “intraoperative, perioperative and early postoperative,” which is a bit circular. I suggest defining once, early in Methods, something like: “perioperative = preoperative fasting/replacement period, intraoperative period, and postoperative period up to X hours/days,” and use that wording consistently.

Search strategy and sources

Strength: you plan a comprehensive, librarian-assisted search in MEDLINE, Embase, CENTRAL, Web of Science, ClinicalTrials.gov and Google Scholar, in 3 languages (English, Spanish, German).

However, the search strings reproduced in the supplement look strongly RCT-oriented (many trial/RCT filters). In the main text you say you will include RCTs, observational studies, and systematic reviews. Please make it explicit that the actual search will not filter out non-randomized designs, or provide a second, broader line to capture non-experimental pediatric surgical fluid studies. Otherwise you risk missing descriptive pediatric cohorts, which in this field are often the only data available.

You say, “searches will be performed up to 2025.” Because the protocol will be published in 2025, specify a month (e.g. “through September 2025”) to increase transparency.

Study selection and data charting

You describe duplicate screening in Covidence with a third reviewer to arbitrate — this is good and standard.

You also say “authors will not be contacted for missing data.” That is acceptable for a scoping review, but please state the consequence (e.g. “the data item will be left blank and reported as ‘not reported’”).

You refer to “Study exclusions will be systematically documented.” Consider adding that you will present a PRISMA-ScR flow diagram.

Data items and synthesis

You list many potential outcomes (fluid balance, hemodynamics, electrolyte disturbances, acid–base, AKI, edema, LOS). For a scoping review, it would help to say you will map which outcomes are reported by which studies, not to analyze them comparatively.

You could pre-specify some stratifications you already mention later (age group: neonate/infant/child/adolescent; type of surgery; ambulatory vs inpatient; fluid type: balanced vs 0.9% saline vs colloid vs dextrose-containing). This will make the descriptive tables easier to follow.

You rightly say no formal risk-of-bias appraisal is planned, “consistent with scoping methodology.” That is acceptable for BMJ Open, but one sentence acknowledging that this limits inferences about effectiveness would be helpful.

Language and repetition

In the abstract/methods there is a duplicated sentence about “Two reviewers will independently screen…” — it appears twice almost verbatim. Please remove one instance.

A light language edit will improve flow (e.g. “we will explore the current evidence…” appears very often).

Ethics and dissemination

You already have ethics approval and you justify it, even though it is not strictly required for a scoping review — that’s fine. Maybe add that protocol registration (e.g. on OSF or Open Science Framework) will be considered; BMJ Open likes visible pre-specification.

Minor editorial points

Ensure consistent naming of the authors (some lines have “AC, GC and MK,” others “AC, GC and MK conceived…” — keep one version).

Keywords: they currently start with “For peer review only…” because of the template — please clean that part.

Check that all references cited (e.g. JBI 2024 manual, Pollock et al. 2023 JBI Evid Synth) are fully formatted per BMJ Open style.

VERSION 1 - AUTHOR RESPONSE

REVIEWER 1 – Dr. Davinia Withington	AUTHOR RESPONSE AND COMMENTS
This is a very useful topic to investigate. However, I am finding it difficult to determine why the authors chose a scoping review vs a systematic review. There seems to be an overlap in the aims between the former and latter type of investigation.	We appreciate this valuable point. Our aim is to map the existing evidence regarding perioperative IV fluid management in pediatric non-cardiac surgery—a field characterized by heterogeneous study designs and limited randomized data.<br><br>A scoping review is therefore appropriate to describe what evidence exists, how it has been studied, and where research gaps lie.<br><br>We added an explicit justification in the Introduction and Methods. Quote: "Given the limited and heterogeneous nature of the available pediatric evidence, a systematic review is currently not yet feasible. The literature spans multiple study designs, varying perioperative periods, mixed age groups, and inconsistent outcome measures, with many practices still extrapolated from adult research. A scoping review is therefore the most appropriate approach to map how perioperative intravenous fluids have been studied in children, to describe the range and characteristics of existing evidence, and to identify gaps that must be addressed before a systematic review can be undertaken."<br><br>Perhaps, in future work, once the range of interventions identified in the literature is clearly mapped, a systematic review could be conducted to evaluate their effectiveness and determine which approaches yield the best outcomes. However, at this stage, our primary objective is to understand what interventions are available in the literature and how they have been studied, which is aligned with the purpose and scope of a scoping review.
A scoping review will provide an overview of the literature, which you do, and identify pertinent concepts and gaps in the current research. The protocol appears to be going beyond this by looking in more depth into outcomes.	Clarified. No. We have clarified in the protocol that the review will not conduct comparative analyses nor undertake a formal risk-of-bias assessment, as these are beyond the scope of a scoping review.<br><br>We also explicitly acknowledge that this limits the ability to draw conclusions about effectiveness but we also highlight that, depending on the gaps identified, a

	subsequent systematic review may be considered.<br><br>We will describe how outcomes are examined in the available literature, but we will not conduct any additional analyses beyond this descriptive mapping.
Also the protocol states that the results may improve perioperative outcomes and decrease complications but it would be difficult to achieve this with a Scoping Review which lacks the Quality Appraisal which would be part of a systematic review.	Corrected. We thank the reviewer for this important observation.<br><br>We agree that a scoping review does not aim to assess the effectiveness of interventions or establish causal relationships, and therefore cannot directly demonstrate improvements in perioperative outcomes or complication rates.<br><br>We have revised the text accordingly to clarify that the purpose of this review is to map and describe the available evidence on perioperative intravenous fluid management in pediatric surgery, identify research gaps, and inform future studies or guideline development, rather than to evaluate clinical impact. Quote from Discussion section “By mapping the existing evidence and identifying gaps in pediatric perioperative fluid management, this review will help clarify areas where further investigation is needed and provide an evidence map of current research, which may inform future systematic reviews and guide priorities for primary studies. This scoping review is not intended to evaluate effectiveness or support clinical recommendations.”
Please clarify which type of investigation is planned and why this was chosen and, if a scoping review, would the investigators move on to a Systematic Review later? The conclusion that a scoping review can contribute to the development of specific clinical guidelines and improve care is overstating the case since a full appraisal of study outcomes is not possible for this study format.	Thank you for this valuable comment. We clarify that the planned investigation is a scoping review, chosen because the existing literature on perioperative intravenous fluid management in pediatric non-cardiac surgery is heterogeneous, extremely limited and largely extrapolated from adult research.<br><br>Only a small number of pediatric studies exist, and they vary widely in design, population, comparators, and reported outcomes. Given this variability, a scoping review is the most appropriate approach to map the breadth of

	available evidence, characterize study designs and outcomes, and identify gaps to inform future research priorities.<br><br>At this stage, a full systematic review is not feasible because the existing evidence base is limited and lacks the methodological consistency needed for formal quality appraisal or quantitative synthesis. Should this scoping review reveal sufficient and comparable pediatric evidence, a subsequent systematic review may be considered; however, this decision will depend entirely on the findings of the current scoping study.<br><br>We fully agree that a scoping review cannot determine effectiveness, appraise study quality, or directly inform clinical guidelines. We have revised the manuscript to ensure we state only that this review will map available evidence and highlight research gaps, without implying that it can improve outcomes or support guideline development.<br><br>We added a paragraph in the introduction section (and also to methods section) to provide more information about this. Quote: "Given the limited and heterogeneous nature of the available pediatric evidence, a systematic review is currently not yet feasible. The literature spans multiple study designs, varying perioperative periods, mixed age groups, and inconsistent outcome measures, with many practices still extrapolated from adult research. A scoping review is therefore the most appropriate approach to map how perioperative intravenous fluids have been studied in children, to describe the range and characteristics of existing evidence, and to identify gaps that must be addressed before a systematic review can be undertaken."

**REVIEWER 2 – Dr. Kevin McCarthy**	**AUTHOR RESPONSE AND COMMENTS**
Thank you for proposing to undertake this work. Fasting guidelines and peri-operative fluid management in children have undergone significant change over the last decade and some historical and dogmatic practices may no longer apply.	Thank you for this thoughtful comment. We agree that perioperative fasting guidelines and fluid management practices in children have evolved considerably in recent years. However, analysing how these guidelines have changed over time or comparing historical versus current practices is beyond the scope and objectives of this scoping review. Addressing such questions would require a different study design and

	methodological approach, likely involving a historical or policy-focused review.<br><br>Our aim is more dedicated **to map the types of intravenous fluids used** in pediatric non-cardiac surgery and to describe how these have been studied in the literature, without evaluating guideline implementation or changes in clinical practice over time.<br><br>Nevertheless, we acknowledge that the variability in clinical practice and evolving recommendations may influence the findings. We will therefore address this point within the limitations of the review and clarify it in the interpretation of results, depending on what the evidence reveals.
1. Neonates are physiologically distinct and should be analysed and reported separately. This has been the practice of large-scale observational studies of paediatric practice (e.g. NECTARINE is a separate study from APRICOT) and the NAP-7 National Audit Project in the UK reported a mortality risk that was an order of magnitude higher in neonates than in children. They are also more prone to hypoglycaemia (and hyperglycaemia as part of a surgical stress responses), therefore considerations and recommendations will be specific to this group in a way that is not generalisable to the other paediatric cohorts (infants, children, and adolescents).	Response: We fully agree. We have pre-specified age-based stratification (neonate, infant, schoolchild, child, adolescent) for data extraction and descriptive synthesis to be extremely clear about.
2. If synthetic colloids are to be included, then coagulopathy and impact upon coagulation should be included as an outcome of interest.	Thanks for this comment. Accepted. We added 'coagulation parameters and bleeding outcomes' as additional data items when synthetic colloids are reported.<br><br>Quote from data analysis: "In accordance with scoping review methodology, we will not compare outcomes across studies. Instead, we will systematically map the outcomes reported in each study—such as fluid balance, hemodynamic parameters, electrolyte or acid–base disturbances, acute kidney injury, edema, coagulopathy or coagulation abnormalities (particularly in studies evaluating synthetic colloids), and length of stay—and describe the methods used to measure these outcomes."

**REVIEWER 3 – Dr. Małgorzata Domagalska**	**AUTHOR RESPONSE AND COMMENTS**
Thank you for submitting this protocol on perioperative intravenous fluid management in pediatric non-cardiac surgery. The topic is well chosen: perioperative fluids in children are still guided largely by extrapolation from adult data, and mapping what actually exists in the literature is valuable and fits well with the aims of a scoping review.<br><br>I have a few suggestions to improve clarity and alignment with PRISMA-ScR/JBI, mostly editorial and clarificatory.	Thanks for this comment.
Rationale and objectives<br>The introduction clearly explains why adult data cannot simply be transferred to children (physiological differences, risk of electrolyte/acid–base disturbances, immature regulation). This is good.<br><br>In the “Research objectives” and “Research questions” sections there is some overlap and partial repetition. Consider condensing into: (a) overarching objective (to map IV fluids used perioperatively in pediatric non-cardiac surgery) and (b) 3–4 explicit scoping questions (types of fluids; benefits/adverse effects; management across perioperative phases; gaps).	We thank the reviewer for her detailed and constructive suggestions, all of which have been incorporated.<br><br>The manuscript was revised to improve alignment with PRISMA-ScR and JBI guidance, clarify methods, and reduce redundancy.<br><br>We have incorporated your recommendations by consolidating the “Research objectives” and “Research questions” sections to avoid overlap and repetition. The manuscript now includes: (a) a single overarching objective focused on mapping the intravenous fluids used perioperatively in pediatric non-cardiac surgery, and (b) a set of **three explicit scoping questions** addressing fluid types, benefits and adverse effects, and management across perioperative phases, as well as remaining evidence gaps. This restructuring improves clarity and alignment with scoping review methodology.
Scope and PCC elements<br>Your PCC is appropriate for a scoping review: P – pediatric patients <18 y with non-cardiac surgery; C – IV fluid management for maintenance/replacement perioperatively; C – hospital or ambulatory surgical setting.<br><br>You exclude critical care, emergency trauma with massive resuscitation, cardioplegia, and hyperosmolar therapeutic fluids — this is sensible, but please say explicitly why these are excluded (i.e. they belong to resuscitation/neurological or cardiac pathways rather than perioperative maintenance).	Thank you for this helpful observation. We have clarified the rationale for these exclusions in the manuscript. These patient groups and clinical situations were excluded because they involve substantially different fluid requirements and physiological dynamics, with large and rapid fluid shifts that are not comparable to perioperative maintenance or replacement therapy.<br><br>Specifically, resuscitation pathways, cardiac bypass perfusion, and neurotherapeutic osmotherapy represent fundamentally

REVIEWER 3 – Dr. Małgorzata Domagalska	AUTHOR RESPONSE AND COMMENTS
	different fluid strategies from perioperative management and therefore fall outside the intended scope of this review.<br><br>Quote from PCC (C section): “These exclusions were applied because resuscitation pathways, cardiac bypass perfusion, and neurotherapeutic osmotherapy represent fundamentally different fluid strategies from perioperative maintenance and replacement, and therefore fall outside the scope of this review.”
You may want to clarify what you will do with peri-induction dextrose-containing solutions in neonates/infants (you mention them, so specify that they will be included when used as part of perioperative fluid strategy).	Clarified. We agree that clarification is needed regarding dextrose-containing solutions administered during induction, particularly in neonates and infants. We have now explicitly stated that peri-induction dextrose-containing fluids will be included when they are used as part of the perioperative intravenous fluid strategy for maintenance or replacement, or when their physiological effects (e.g., glycemic stability) are evaluated in the context of perioperative fluid management.<br><br>We have updated the Methods section to clarify this inclusion criteria: Quote “Additionally, dextrose-containing solutions administered during induction, particularly in neonates and infants, will be included when they are used as part of the perioperative intravenous fluid strategy (for maintenance or replacement) or when their physiological effects (e.g., glycemic stability, acid–base status, electrolyte balance) are evaluated in the context of perioperative fluid management.”
Perioperative time windows<br>You often write “perioperative (pre/intra/early postoperative)” but in other places you say “intraoperative, perioperative and early postoperative,” which is a bit circular. I suggest defining once, early in Methods, something like: “perioperative = preoperative fasting/replacement period, intraoperative period, and postoperative period up to X hours/days,” and use that wording consistently.	Thank you for this suggestion. We have incorporated a single, clear definition early in the Methods section and have applied this terminology consistently throughout the protocol.<br><br>Quote from Methods section.<br><br>Definition of perioperative period: For the purposes of this review, the term perioperative refers to the continuum that includes:<br>(1) the preoperative fasting and replacement period,<br>(2) the intraoperative period, and

REVIEWER 3 – Dr. Małgorzata Domagalska	AUTHOR RESPONSE AND COMMENTS
	(3) the early postoperative period, defined as the first 24 hours after completion of surgery. This definition will be applied consistently throughout the review.
Search strategy and sources<br>Strength: you plan a comprehensive, librarian-assisted search in MEDLINE, Embase, CENTRAL, Web of Science, ClinicalTrials.gov and Google Scholar, in 3 languages (English, Spanish, German).<br>However, the search strings reproduced in the supplement look strongly RCT-oriented (many trial/RCT filters). In the main text you say you will include RCTs, observational studies, and systematic reviews. Please make it explicit that the actual search will not filter out non-randomized designs, or provide a second, broader line to capture non-experimental pediatric surgical fluid studies. Otherwise you risk missing descriptive pediatric cohorts, which in this field are often the only data available.	Thank you for this comment. We have clarified that the final search strategy will not use filters that restrict results to randomized trials. The search will be conducted without design limitations to ensure that observational and descriptive pediatric cohorts which constitute much of the available evidence are captured. The supplementary search strings have been updated accordingly.<br><br>In addition, we will receive support for the entire search process from a professional librarian, and the detailed search strategy is available in the Supporting Information, developed in collaboration with the Erasmus MC University Medical Library (The Netherlands).
You say, "searches will be performed up to 2025." Because the protocol will be published in 2025, specify a month (e.g. "through September 2025") to increase transparency.	Thank you for this suggestion. We agree that specifying this will improve transparency.<br>We have revised the methods section to intricate the precise month.
Study selection and data charting<br>You describe duplicate screening in Covidence with a third reviewer to arbitrate — this is good and standard.<br><br>You also say "authors will not be contacted for missing data." That is acceptable for a scoping review, but please state the consequence (e.g. "the data item will be left blank and reported as 'not reported'").<br>You refer to "Study exclusions will be systematically documented." Consider adding that you will present a PRISMA-ScR flow diagram.	Completed. We have added that missing data will be recorded as *"not reported"* and fields will remain blank when information cannot be obtained from the published source. We also now state that study exclusions will be systematically documented and presented in a PRISMA-ScR flow diagram. The duplicate screening and arbitration process remains unchanged.
Data items and synthesis<br>You list many potential outcomes (fluid balance, hemodynamics, electrolyte disturbances, acid–base, AKI, edema, LOS). For a scoping review, it would help to say you will map which outcomes are reported by which studies, not to analyze them comparatively.<br><br>You could pre-specify some stratifications you already mention later (age group:	Thank you for these insightful suggestions. We have clarified in the Methods section that, consistent with scoping review methodology, we will *map* which outcomes are reported by each study rather than analyze or compare outcomes across studies.<br><br>We have also pre-specified the descriptive stratifications that will be used during data charting, including age group (neonate, infant,

REVIEWER 3 – Dr. Małgorzata Domagalska	AUTHOR RESPONSE AND COMMENTS
neonate/infant/child/adolescent; type of surgery; ambulatory vs inpatient; fluid type: balanced vs 0.9% saline vs colloid vs dextrose-containing). This will make the descriptive tables easier to follow.<br><br>You rightly say no formal risk-of-bias appraisal is planned, "consistent with scoping methodology." That is acceptable for BMJ Open, but one sentence acknowledging that this limits inferences about effectiveness would be helpful.	child, adolescent), type of surgery, surgical setting (ambulatory vs inpatient), and type of intravenous fluid (balanced crystalloids, 0.9% saline, colloids, and dextrose-containing solutions).<br><br>In addition, we have added a statement acknowledging that the absence of a formal risk-of-bias appraisal limits any inference regarding the effectiveness or safety of the interventions described in the included studies. Quote from data analysis "Consistent with scoping review methodology, we will not perform formal risk-of-bias appraisal. We acknowledge that the absence of quality assessment limits the ability to draw conclusions on the effectiveness or comparative safety of the interventions described."
Language and repetition<br>In the abstract/methods there is a duplicated sentence about "Two reviewers will independently screen…" — it appears twice almost verbatim. Please remove one instance. A light language edit will improve flow (e.g. "we will explore the current evidence…" appears very often).	We have removed the duplicated sentence and edited the corresponding sections to improve flow and reduce repetition. We appreciate the suggestion.
Ethics and dissemination<br><br>You already have ethics approval and you justify it, even though it is not strictly required for a scoping review — that's fine. Maybe add that protocol registration (e.g. on OSF or Open Science Framework) will be considered; BMJ Open likes visible pre-specification.	Thank you for this important suggestion.<br><br>Although ethical approval is not required for a scoping review, we have retained the institutional approval as part of internal policy requirements.<br><br>In response to your comment, we have now added that the protocol will be registered in an open-access platform (such as the Open Science Framework) to ensure transparency, pre-specification, and public accessibility, in line with BMJ Open's preference for visible protocol registration.<br><br>Quote: "In addition, protocol registration (e.g., on the Open Science Framework) will be considered to enhance transparency and reproducibility."
Minor editorial points<br><br>Ensure consistent naming of the authors (some lines have "AC, GC and MK," others "AC, GC and MK conceived…" — keep one	Done. We have ensured consistent naming of the authors throughout the manuscript, removed the "For peer review only…" text from the keywords, and verified that all references—including the JBI 2024 manual

REVIEWER 3 – Dr. Małgorzata Domagalska	AUTHOR RESPONSE AND COMMENTS
version).<br><br>Keywords: they currently start with “For peer review only…” because of the template — please clean that part.<br><br>Check that all references cited (e.g. JBI 2024 manual, Pollock et al. 2023 JBI Evid Synth) are fully formatted per BMJ Open style.	and Pollock et al. (2023) JBI Evidence Synthesis—are fully formatted according to BMJ Open style.

VERSION 2 - REVIEW

**Reviewer**	**3**
**Name**	**Reysner, Malgorzata**
**Affiliation**	**Poznan University of Medical Sciences**
**Date**	**25-Dec-2025**
**COI**	

This is a strong and carefully developed scoping review protocol on a clinically important topic with clear relevance to pediatric anesthesia and surgery. The methodological approach is appropriate, transparent, and aligned with PRISMA-ScR and JBI guidance. The authors appropriately avoid overinterpretation and clearly frame the review as an evidence-mapping exercise rather than an effectiveness assessment.

Major revisions are suggested to improve clarity and transparency.

1. Clarify reporting vs conduct terminology.

Please ensure consistent wording throughout the manuscript to clearly distinguish between methodological conduct (guided by JBI methodology) and reporting (guided by PRISMA-ScR). Avoid statements implying that PRISMA-ScR guides the conduct of the review.

2. Specify the search end date precisely.

Please specify the exact month and year up to which the searches will be conducted (e.g. “searches will be performed through October 2025”) to improve transparency and reproducibility.

3. Search strategy clarity (non-RCT evidence)

Although this is addressed in the text, please ensure it is clearly stated in the Methods section that the final operational search will not apply RCT-only filters, in order to capture observational and descriptive pediatric studies, which are common in this field.

4. Streamline objectives and research questions

There is minor overlap between the “Research objectives” and “Research questions” sections. Consider consolidating these into one overarching objective followed by clearly delineated scoping questions.

5. Consistency in perioperative time-point terminology

Please ensure consistent use of the predefined perioperative phases (preoperative fasting/replacement, intraoperative, early postoperative up to 24 hours) throughout the manuscript to avoid circular or interchangeable phrasing.

6. Handling of missing data

Please explicitly state that when required information is not available in a publication, the data item will be recorded as “not reported” and left blank in the data charting tables.

7. PRISMA-ScR flow diagram

Please explicitly state that the study selection process will be presented using a PRISMA-ScR flow diagram, even though this is a protocol.

8. Risk-of-bias clarification

While it is appropriate not to perform formal risk-of-bias assessment in a scoping review, please retain a brief explicit statement acknowledging that this limits inference regarding effectiveness or comparative safety.

9. Minor language and repetition edits

Please remove duplicated sentences (e.g. repeated descriptions of duplicate screening) and consider light language editing to reduce repetition of phrases such as “we will explore the current evidence”.

10. Keywords clean-up

Please remove template artefacts (e.g. “For peer review only”) from the keyword list and ensure all keywords are formatted according to BMJ Open style.

11. Protocol registration

Consider explicitly stating whether protocol registration (e.g. Open Science Framework) is planned or underway.

VERSION 2 - AUTHOR RESPONSE

Reviewer: 3<br>Dr. Malgorzata Reysner, Poznan University of Medical Sciences<br><br>Comments to the Author:<br>This is a strong and carefully developed scoping review protocol on a clinically important topic with clear relevance to pediatric anesthesia and surgery. The methodological approach is appropriate, transparent, and aligned with PRISMA-ScR and JBI guidance. The authors appropriately avoid overinterpretation and clearly frame the review as an evidence-mapping exercise rather than an effectiveness assessment.<br>Major revisions are suggested to improve clarity and transparency.<br><br>1. Clarify reporting vs conduct terminology. Please ensure consistent wording throughout the manuscript to clearly distinguish between methodological conduct (guided by JBI methodology) and reporting (guided by PRISMA-ScR). Avoid statements implying that PRISMA-ScR guides the conduct of the review.	thanks for your comment, We revised the wording in the Methods and Analysis section to clearly distinguish between methodological conduct and reporting. The conduct of the scoping review is described as being guided by the Joanna Briggs Institute methodology, while reporting is specified as adhering to the PRISMA-ScR guidelines.
2. Specify the search end date precisely. Please specify the exact month and year up to which the searches will be conducted (e.g. “searches will be performed through October 2025”) to improve transparency and reproducibility.	Thank you for this suggestion. We have specified the exact search end date in the Search Strategy section, indicating that all searches will be performed up to October 2025 to enhance transparency and reproducibility.
3. Search strategy clarity (non-RCT evidence)<br>Although this is addressed in the text, please ensure it is clearly stated in the Methods section that the final operational search will not apply RCT-only filters, in order to capture observational and descriptive pediatric studies, which are common in this field.	We have stated in the Methods section (Search Strategy) that the final operational search will not apply randomized trial–only filters or methodological limits. This clarification ensures the inclusion of observational and descriptive pediatric studies, which constitute a substantial

	proportion of the available evidence in this field.
4. Streamline objectives and research questions<br>There is minor overlap between the “Research objectives” and “Research questions” sections. Consider consolidating these into one overarching objective followed by clearly delineated scoping questions.	Done
5. Consistency in perioperative time-point terminology<br>Please ensure consistent use of the predefined perioperative phases (preoperative fasting/replacement, intraoperative, early postoperative up to 24 hours) throughout the manuscript to avoid circular or interchangeable phrasing.	Thank you for this comment. We have standardized the terminology used to describe perioperative time points throughout the manuscript. The predefined phases are now consistently referred to as the preoperative fasting/replacement period, intraoperative period, and postoperative period up to 24 hours across all relevant sections, avoiding interchangeable or circular phrasing.
6. Handling of missing data<br>Please explicitly state that when required information is not available in a publication, the data item will be recorded as “not reported” and left blank in the data charting tables.	We have clarified in the Data Extraction and Management section that when required information is not available in a publication, the corresponding data item will be recorded as “not reported” and left blank in the data charting tables.
7. PRISMA-ScR flow diagram<br>Please explicitly state that the study selection process will be presented using a PRISMA-ScR flow diagram, even though this is a protocol.	We have stated in the Methods section that the study selection process will be presented using a PRISMA-ScR flow diagram, even though this manuscript reports a scoping review protocol.
8. Risk-of-bias clarification<br>While it is appropriate not to perform formal risk-of-bias assessment in a scoping review,	We have explicitly retained a statement in the Methods section acknowledging that the

please retain a brief explicit statement acknowledging that this limits inference regarding effectiveness or comparative safety.	absence of formal risk-of-bias assessment limits the ability to draw conclusions regarding effectiveness and comparative safety, consistent with the objectives of a scoping review.
9. Minor language and repetition edits<br>Please remove duplicated sentences (e.g. repeated descriptions of duplicate screening) and consider light language editing to reduce repetition of phrases such as “we will explore the current evidence”.	Done
10. Keywords clean-up<br>Please remove template artefacts (e.g. “For peer review only”) from the keyword list and ensure all keywords are formatted according to BMJ Open style.	We have cleaned up the keyword list by removing template artefacts and formatting all keywords according to BMJ Open style.
11. Protocol registration<br>Consider explicitly stating whether protocol registration (e.g. Open Science Framework) is planned or underway.	We have stated in the Ethics and Dissemination section that protocol registration on an open-access platform such as the Open Science Framework will be considered to enhance transparency and reproducibility
Reviewer: 3<br>Competing interests: NA	
